# Female genital mutilation and sexual behaviour by marital status among a nationally representative sample of Nigerian women

**DOI:** 10.1186/s12978-022-01379-w

**Published:** 2022-04-07

**Authors:** Babatunde Adelekan, Yusuf Olushola Kareem, Zubaida Abubakar, Karima Bungudu, Adewale Aderemi, Erika Goldson, Ulla Mueller, Sanni Yaya, Adesegun Fatusi

**Affiliations:** 1 United Nations Population Fund, Abuja, Nigeria; 2 Osun State University, Osogbo, Osun State Nigeria; 3 Faculty of Social Sciences, School of International Development and Global Studies, University of Ottawa, 120 University Private, Ottawa, ON K1N 6N5 Canada; 4 The George Institute for Global Health, Imperial College London, London, UK; 5 University of Medical Sciences, Ondo City, Ondo State Nigeria

**Keywords:** Sexual and reproductive health, Sexual behaviour, Female genital mutilation, Nigeria

## Abstract

**Background:**

Female Genital Mutilation (FGM) is believed to have a negative effect on sexual and reproductive health but the evidence from nationally representative sample in high-burdened countries like Nigeria is scarce. This study explored the association between FGM and sexual behaviour in a nationally representative sample of Nigerian women.

**Methods:**

A secondary data analysis was conducted using the Nigeria Demographic Health Survey conducted in 2013 and 2018 among women aged 15–49 years. The descriptive summaries of respondent characteristics by marital status were presented using frequencies and percentages. The proportion and 95% Confidence Interval (CI) of circumcision by sexual behaviour characteristics were computed. A multivariable log-binomial logistic regression was used to determine the association between sexual behaviour and female circumcision while adjusting for other covariates. All analyses were performed using Stata 15.1 (StataCorp, College Station, TX, USA) at the 0.05 level of significance.

**Results:**

The proportion of circumcised women was 38.6% among those who were ever-married and 32.4% among those unmarried. There were no statistically significant relationship between circumcision status and sexual behaviour among women who were unmarried. However, circumcised women who were ever married had 18% higher risk of having contracted sexually transmitted disease in the last 12 months preceeding the survey and 10% higher risk of engaging in pre-marital sex compared to ever married women who were uncircumcised after adjusting for other covariates. However, the risk of having multiple sexual partners in the last 12 month among uncircumcised ever married women was lower (aRR = 0.80; 95% CI: 0.66–0.97) in the adjusted model.

**Conclusion:**

Circumcision is not associated with positive sexual behavioural outcomes including delay in sexual debut, virginity and marital fidelity, although there exists some perception behind increasing FGM in Nigeria including prevention of premarital sex and ensuring marital fidelity. While we strongly discourage FGM in all its form, we assert the need for alternative health promoting community measures to address these inherent sexual perceptions toward eliminating FGM and improving sexual and reproductive health across population groups.

**Supplementary Information:**

The online version contains supplementary material available at 10.1186/s12978-022-01379-w.

## Plain English Summary

Female genital mutilation (FGM) recognized internationally as a violation of human right, refers to all procedures that involve partial or complete removal or other injury to the female genital organs for non-medical reasons. About 200 million girls and women in 30 countries mainly in Africa, Middle East and Asia have been subjected to FGM. Almost 22% of the estimated 68 million girls and women that are at the risk of being cut between 2015 and 2030 will be Nigerians.

FGM is deeply entrenched within socio-cultural beliefs around preserving a girl's purity, and controlling women's sexuality or chastity. It is also believed that FGM protects girl’s virginity, prevents marital infidelity and is necessary to ensure male partners and/or husbands get better sexual satisfaction. However, studies on the association between women sexual behaviour (sexual debut, pre-marital sex, multiple sexual partners, STDs, lifetime sexual partner, number of unions) and FGM is limited in countries where FGM is prevalent. This study utilized pooled datasets from the Nigerian Demographic Health Survey conducted in 2013 and 2018 to explore the association between FGM and sexual behaviour among women of reproductive age.

The findings revealed that there is no association between sexual behaviour and FGM among unmarried women. Circumcised ever-married women were more likely to contract sexually transmitted diseases and engage in pre-marital sex than those who were not circumcised. However, circumcised ever married women were less likely to have multiple sexual partner in the last 12 months preceding the survey. This study disproves the assertion that FGM could control women’s sexual behaviour.

**Supplementary Information:**

The online version contains supplementary material available at 10.1186/s12978-022-01379-w.

## Background

Female circumcision, also known as Female Genital Mutilation (FGM) is a global women and girls’ health challenge and a form of gender-based violence. It is a gross violation of women’s rights. FGM affects at least 200 million women and girls across 30 countries and more than three million new cases occur every year [1]. Female Genital Mutilation (FGM) is described as all procedures involving partial or total removal of the external female genitalia or other injury to the female genital organs, whether for cultural or other non-therapeutic reasons [1]. Female genital mutilation is an age-long practice that transcends religion, geography, and socioeconomic status [2]. Despite considerable global attention, the rate of decrease in the prevalence of FGM is lower than the rate of population growth, and therefore the number of girls and women undergoing and at risk of FGM continues to rise [3]. Indeed, for Nigeria to meet it’s commitment to the Sustainable Development Goal (SDG) 5.3 that targets the elimination of all harmful practices including female genital mutilations as part of the focus on achieving gender equality and empowering all women and girls [4], FGM myths and misconceptions must be addressed.

Nigeria has an estimated 20 million women and girls with history of FGM [5], and ranks third globally in terms of burden of FGM [6]. Nigeria contributes about a tenth of the global burden of FGM, despite its population being only about three percent of the global population. While there have been significant efforts aimed at eliminating FGM in Nigeria with laws promulgated at state and national levels and some community-based interventions mounted. The progress against FGM has been fairly slow, with the Multiple Indicator Cluster Survey reporting a reduction in prevalence from 27% in 2011 to 18.4% in 2017 among women aged 15–49 years [7], while it decreased from 25% in 2013 to 20% in 2018 according to the Nigerian Demographic Health survey (NDHS) [8, 9]. In 2013, up to a third of adult female Nigerians still supported the continuation of FGM [8].

FGM has been associated with various sexual and reproductive health challenges in the literature, including sexual dysfunction, painful intercourse, infertility, clitoral cyst, reduced sexual desire and satisfaction, and recurrent urinary tract infection [10, 11]. However, recent work on the sexual experiences of women with FGM is relatively lacking in FGM burden countries including Nigeria. The latest study known to the authors on this issue utilized 2008 NDHS data to investigate the relationship between FGM and sexual behavior—assessed using age at sexual initiation and number of lifetime partner [12]. However, the NDHS 2008 definition of FGM excludes some uncategorized type of FGM based on the WHO 2008 definition. This study explored the association between FGM and sexual behavior in a nationally representative sample of Nigerian women using pooled datasets from the 2013 and 2018 NDHS [8, 9].

## Materials and methods

### Data source and sampling strategy

This study utilized the individual women recode datasets of women aged 15–49 years from the two most recent NDHS conducted in 2013 and 2018 NDHS. The definition of female genital mutilation and other related questions were comparable for the year 2013 and 2018. NDHS is a nationally representative survey that uses stratified multi-stage cluster sampling and quantitative data collection with standardized questions to provide relevant population and health estimates at the national, regional and at the state level. The 36 States and Federal Capital Territory (FCT) were stratified into urban and rural areas. Then, the first stage is the selection of clusters, also known as enumeration areas (EAs) and the next stage involves the selection of individuals in the households selected for the survey. The response rate for the 2013 and 2018 NDHS was 99 percent and the sampling weights were adjusted for household and individual non-response. Although, the sampling weight are usually normalized to make the total number of unweighted cases to be equal to the number of weighted cases at the national level, the sampling weights were denormalized in this study before the datasets were pooled for a valid estimation. The weighted pooled sample size included 29,724 ever married and 8549 unmarried women aged 15–49 years.

### Outcome variables

We assessed sexual behavior using six indicator variables: (i) age of sexual debut, classified as early—if respondent initiated sex before age 15 or latter (< 15 vs ≥ 15 years); (ii) had multiple sexual partner in the last 12 months preceding the survey (no vs yes); (iii) the number of lifetime sexual partners (none/single vs multiple); (iv) history of sexually transmitted disease (STD) in the last 12 months before the survey (no vs yes); (v) history of premarital sex among those married (no vs yes) (vi) and number of marital unions (once vs more than once) were included for respondents who were married.

### Independent variables

The main independent variable was whether the respondent was circumcised or not; other covariates considered were respondents age group (15–19, 20–24, 25–29, 30–34, 35–39, 40–44 and 45–49), level of education (none, primary, secondary and tertiary), wealth quintiles (poorest, poorer, middle, richer and richest), current work status (no vs yes), place of residence (urban vs rural), religion (Christians, Muslims and others), ethnicity (Fulani, Hausa, Igbo, Yoruba and others), region (North Central, North East, North West, South East, South South and South West) and age at marriage were considered for respondents who were married.

### Statistical techniques

The sampling weight for the datasets were denormalized and adjusted for the population size of women aged 15–49 extracted from World Bank staff estimates [13], then the datasets were then pooled together for the analysis. All statistical analyses were weighted and adjusted for the complex survey design and performed using Stata 15.1 (StataCorp, College Station, TX, USA). To explicitly identify possible sexual behavioural characteristics, the analysis were done seperately based on marital status. Women who were currently in a union or formerly in a union were classified as ever married while those who were single were classified as never married.

The descriptive summaries of respondent characteristics by marital status were presented using frequencies and percentages while the only continuous variable age at marriage was summarised using the median and inter-quartile range due to the skewness of the variable. An assessment of multicollinearity showed no significant correlation between the various sexual behaviours. Then, a multivariable log-binomial model was used to investigate the association between FGM and sexual behaviour while simultaneously adjusting for the covariates; the adjusted relative risk (aRR) and 95% confidence intervals were presented and statistical inferences were interpreted at the 0.05 level of significance.

## Results

### Descriptive summaries

The number of ever married respondents were 29,724 (78%) while 8,549 respondents (22%) were single (Table 1). The median age at first marriage for those ever-married was 17 years (interquartile range [IQR] 15–21). About one in four of the ever-married women and one in 25 among those unmarried initiated sex before age 15. A higher proportion (35.1%) of unmarried women of reproductive age compared to 2.4% of respondents who were married had multiple sexual partners in the last 12 months before the survey. Also, 35.3% of women who were ever married and 24.5% of unmarried women had multiple lifetime sexual partner.

**Table 1:** Descriptive summaries (outcome variables) of respondents by marital status

Characteristics	Ever married (N = 29,724)	Never married (N = 8549)
	Frequency (%)	Frequency (%)
Outcome variables		
Sexual debut		
< 15 years	6959 (23.4)	321 (3.8)
≥ 15 years	22,765 (76.6)	8228 (96.2)
Had multiple sexual partners in the last 12 months		
No	29,004 (97.6)	5552 (64.9)
Yes	720 (2.4)	2997 (35.1)
Lifetime number of sexual partners		
≤ 1 (none/single)	19,233 (64.7)	6451 (75.5)
> 1 (multiple)	10,491 (35.3)	2098 (24.5)
Had STD in the last 12 months		
No	28,155 (94.7)	8249 (96.5)
Yes	1569 (5.3)	300 (3.5)
Had pre-marital sex/virginity status^a^		
No	20,043 (67.4)	4548 (53.2)
Yes	9681 (32.6)	4001 (46.8)
Number of unions		
Once	26,595 (89.5)	NA
More than once	3129 (10.5)	NA

*NA* not applicable

^a^Virginity status used for unmarried women who had not initiated sexual intercourse

Similarly, 67.4% of ever married women never had premarital sex while 53.2% of women who are still single had never had sexual intercourse and are virgins. Almost one in ten of the ever-married women have had more than one union.

About half of the unmarried women were aged 15–19 but with the smallest percentage (5.8%) among those ever-married; while other age group intervals were almost evenly distributed. The percentage of respondents with secondary and higher level of education was lower among ever-married women compared to those unmarried, while the percentage of ever-married women with no formal education was higher (41.8% vs 5.8%) compared to unmarried women.

Although, the wealth quintiles are evenly distributed among the ever-married women, a higher proportion of ever-married women were in the poorest and poorer quintiles compared to those unmarried. A higher proportion of women who were ever-married are currently working (74.0% vs 44.7%) compared to those unmarried. Similarly, a higher proportion of the ever-married woman live in the urban areas (55.7% vs 34.3%) compared to unmarried women. More than half of the ever-married women practice Islam religion while seven(7) in ten (10) of those unmarried were Christians. The major ethnic groups among those ever-married was Hausa and Yoruba for those who were unmarried. Also, a higher proportion of those ever-married or unmarried were from the North western region (34.2%) or  South South (76.3%) region respectively (Table 2).

**Table 2:** Descriptive summaries (independent and co-variates) of respondents by marital status

Characteristics	Ever Married (N = 29,724)	Never Married (N = 8549)
	Frequency (%)	Frequency (%)
Main independent variable (circumcised)		
No	18,247 (61.4)	5780 (67.6)
Yes	11,477 (38.6)	2769 (32.4)
Other covariates		
Age		
15–19	1710 (5.8)	4337 (51.1)
20–24	4059 (13.7)	2240 (26.2)
25–29	5786 (19.4)	1146 (13.4)
30–34	5281 (17.8)	471 (5.5)
35–39	4951 (16.7)	201 (2.4)
40–44	3960 (13.3)	74 (0.9)
45–49	3977 (13.4)	51 (0.6)
Level of education		
No formal education	12,411 (41.8)	492 (5.8)
Primary	5614 (18.9)	652 (7.6)
Secondary	8647 (29.1)	5645 (66.0)
Higher	3051 (10.3)	1760 (20.6)
Wealth quintiles		
Poorest	6022 (20.3)	456 (5.3)
Poorer	5646 (19.0)	840 (9.8)
Middle	5256 (17.7)	1594 (18.7)
Richer	6063 (20.4)	2412 (28.2)
Richest	6736 (22.7)	3248 (38.0)
Currently working		
No	7716 (26.0)	4729 (55.3)
Yes	22,008 (74.0)	3820 (44.7)
Place of residence		
Urban	13,180 (44.3)	5618 (65.7)
Rural	16,544 (55.7)	2931 (34.3)
Religion		
Christians	12,586 (42.3)	6021 (70.4)
Muslims	16,921 (56.9)	2478 (29.0)
Others	217 (0.7)	50 (0.6)
Ethnicity		
Fulani	2025 (6.8)	137 (1.6)
Hausa	9798 (33.0)	1042 (12.2)
Igbo	3007 (10.1)	1022 (12.0)
Yoruba	4595 (15.4)	2537 (29.7)
Others	10,299 (34.7)	3814 (44.6)
Region		
North Central	2364 (8.0)	796 (9.3)
North East	4807 (16.2)	782 (18.5)
North West	10,159 (34.2)	1094 (12.8)
South East	3675 (12.4)	2055 (55.3)
South South	3487 (11.7)	1797 (76.3)
South West	5232 (17.6)	2025 (23.7)
Age at marriage (continuous) median (IQR)	17 (15–21)	NA

NA not applicable

### Proportion of women circumcised by sexual behavioural characteristics

The proportion and 95% confidence interval of women circumcision status by their sexual behavioural characteristics were computed separately among ever married and those unmarried (Table 3). The proportion of those cut was 38.6% among ever married women (95% CI: 36.8–40.4) and 32.4% (95% CI: 30.2–34.7) among those unmarried. The analysis showed no statistical relationship between circumcision status and sexual behaviour among women who were unmarried. However, for women who were ever married; the proportion of women cut were lower among those with early sexual debut (34.8% vs 39.8%; p < 0.001), those with single lifetime partner (35.7% vs 37.8%; p < 0.001) and among those women who had no pre-marital sex (35.1% vs 44.0%; p < 0.001).

**Table 3:** Proportion of circumcision with sexual behavioural characteristics by marital status

Characteristics	Women circumcised	
	Proportion (95% CI)	Proportion (95% CI)
	Ever married	Never married
Sexual debut	***p* < *0.001***	*p* = *0.987*
< 15 years	34.8 (32.1–37.5)	32.5 (25.2–40.6)
≥ 15 years	39.8 (38.0–41.6)	32.4 (30.2–34.7)
Had multiple sexual partners in the last 12 months	*p* = *0.158*	*p* = *0.450*
No	38.5 (36.7–40.4)	32.9 (30.3–35.5)
Yes	42.1 (37.1–47.2)	31.5 (28.5–34.8)
Lifetime number of sexual partners	***p* < *0.001***	*p* = *0.392*
≤ 1 (none/single)	35.7 (33.6–37.8)	32.8 (30.4–35.3)
> 1 (multiple)	44.0 (41.9–46.0)	31.1 (27.5–34.9)
Had STD in the last 12 months	*p* = *0.178*	*p* = *0.632*
No	38.5 (36.7–40.3)	32.5 (30.3–34.7)
Yes	41.1 (37.1–45.3)	30.4 (22.5–39.7)
Had pre-marital sex/virginity status	***p* < *0.001***	*p* = *0.569*
No	35.1 (33.1–37.1)	31.9 (29.3–34.7)
Yes	46.3 (44.1–48.5)	32.9 (30.1–35.9)
Number of unions	*p* = *0.384*	
Once	38.7 (36.9–40.6)	NA
More than once	37.6 (35.0–40.4)	NA

NA not applicable

### The crude and adjusted multivariable log-binomial regression model

The unadjusted log-binomial regression model of the association between sexual behaviour and female circumcision is presented in Table 4 and the model which adjusted for other covariates is presented in Table 5. The full multivariable adjusted regression model can be found as (Additional file 1, 2: Tables S1, S2). The unadjusted model revealed no statistically significant association between sexual behaviour and female circumcision among unmarried women. Ever married women who were circumcised were 1.05 times more likely (95% CI: 1.03–1.08) to initiate sexual intercourse at age 15 or older. Similarly, ever-married circumcised women were more likely to have more than one lifetime sexual partner (RR = 1.06; 95% CI: 1.04–1.08) and also with a higher likelihood (RR = 1.37; 95% CI: 1.28–1.46) of having pre-marital sex.

**Table 4:** Unadjusted log-binomial regression of the association between sexual behaviour and female circumcision by marital status

Variable	Sexual debut	Multiple sexual partner	Number of Lifetime sexual partner	Had STD	Pre-marital sex/virginity status	Number of unions
	RR(95% CI)	RR(95% CI)	RR(95% CI)	RR (95% CI)	RR (95% CI)	RR (95% CI)
Ever married						
Circumcised	***p* < *0.001***	*p* = *0.158*	***p* < *0.001***	*p* = *0.178*	***p* < *0.001***	p = 0.385
No	Reference	Reference	Reference	Reference	Reference	Reference
Yes	**1.05 (1.03–1.08)**	1.15 (0.95–1.41)	**1.06 (1.04–1.08)**	1.11 (0.95–1.29)	**1.37 (1.28–1.46)**	1.00 (0.99–1.00)
Never married						
Circumcised	*p* = *0.987*	*p* = *0.451*	*p* = *0.391*	*p* = *0.632*	*p* = *0.568*	
No	Reference	Reference	Reference	Reference	Reference	
Yes	1.00 (0.99–1.01)	0.96 (0.87–1.07)	0.99 (0.96–1.02)	0.91 (0.63–1.33)	1.02 (0.94–1.11)	

Estimates in bold shows significance at p < 0.05

*RR* relative risk

**Table 5:** Multivariable log-binomial regression of the association between sexual behaviour and female circumcision by marital status

Variable	Sexual debut	Multiple sexual partner	Number of lifetime sexual partner	Had STD	Pre-marital sex/virginity status	Number of unions
	aRR (95% CI)	aRR (95% CI)	aRR (95% CI)	aRR (95% CI)	aRR(95% CI)	aRR(95% CI)
Ever married						
Circumcised						
No	Reference	Reference	Reference	Reference	Reference	Reference
Yes	0.97 (0.94–1.02)	**0.80 (0.66–0.97)**	1.01 (0.99–1.02)	**1.18 (1.00–1.38)**	**1.10 (1.05–1.15)**	1.00 (0.99–1.01)
Never married						
Circumcised						
No	Reference	Reference	Reference	Reference	Reference	
Yes	1.00 (0.99–1.01)	0.99 (0.90–1.10)	1.02 (0.94–1.10)	0.90 (0.63–1.29)	1.02 (0.95–1.09)	

*aRR* adjusted relative risk

The model was adjusted for age, education, wealth, current work status, place of residence, religion, ethnicity, region and age at marriage for married participants only; estimates in bold shows significance at p < 0.05

The log-binomial model was adjusted for respondent age, age at marriage, level of education, wealth status, current work status, place of residence, religion, ethnicity and region. Similar to the crude analysis, there was no statistically significant association between sexual behaviour and female circumcision among women who were unmarried. The findings revealed that ever-married circumcised women had 18% higher risk of having STD in the last 12 months before the survey and 10% higher risk of pre-marital sex compared to ever married women who were not circumcised. However, the risk of multiple sexual partners in the last 12 months was lower (aRR = 0.80; 95% CI: 0.66–0.97) among those who were circumcised after adjusting for other covariates.

## Discussion

The finding in this study that there was no association between female genital mutilation and early sexual debut is in consonance with the study that found no relationship between female circumcision and age at first intercourse in Nigeria and Kenya [14], and disproves the assertion that circumcised women were more likely to initiate first sexual experience compared to uncircumcised women [15]. This study also revealed that ever married women who were mutilated were at lower risk of having multiple sexual partners in contrast to a study in Sierra Leone that showed an increased risk of multiple sexual partners among circumcised women [16].

Our study disproves one of the cultural beliefs deeply held to which also forms a basis for the practice of FGM, which is supposedly aimed at reducing promiscuity and ensuring acceptable sexual behavior including virginity and fidelity [17, 18]. Therefore, there is need to understand the underlying complexities of this cultural belief in order to design successful, culturally acceptable, and correctly targeted FGM eradication campaigns [18, 19].

Increased risk of STDs among circumcised ever married women in this study agrees with the assertion that circumcised women have increased risk of recurrent urinary tract infection [10, 11]. Similarly, previous studies have also revealed that circumcised women might suffer sexual dysfunction and painful sexual intercourse [2, 11, 20–22] and were significantly unlikely to experience sexual desire and satisfaction than uncircumcised women [11] as arousal, lubrication, orgasm and satisfaction affected sexual experience in circumcised women compared to uncircumcised women [10].

Many initiatives, approaches and efforts that have been initiated to eradicate the practice of FGM including increasing legislation [11, 18] and messaging that inform on the adverse health effects are without much success [23–25]. Exposure of negative health consequences or criminalization of the practise often time results in the medicalization of FGM and introduction of various methods to continue the practise secretly [26]. Previous efforts and interventions by organizations have failed because they are thought to be driven by the motive to westernize societies and are based on unproven and misleading information [22] to give Government the impetus to legislate against FGM.

To encourage behaviour change, education about health and rights should be accompanied by discussing and debating the underlying reasons for the practice which can help to proffer desire for change emanating from the communities [27]. Webb and his colleague put it succinctly when they said that, “Any health education initiative which at best could be conceived as culturally hostile is doomed unless it occurs in the context of an overall strategy to improve the health and social welfare of the population as a whole” [28].

Also, the reduction in the practice of FGM is not commensurate with the efforts put in place so far to discourage it because traditional and cultural beliefs are difficult to change especially without addressing the underlying gender inequalities perpetuating the practice. First, there is need to develop home grown strategies and interventions against FGM that considers the cultural and traditional orientation of its practitioners to enable positive behavioural change based on trust and acceptability [22]. Second, there is need to see if the tenacity and persistence of FGM practice and the reluctance for change in communities who practice FGM/C [29] is due to insufficient scientific evidence against it which our study has tried to address.

### Strengths and limitations

This study used nationally representative datasets and robust statistical technique to explore the association between FGM and sexual behaviour among women aged 15–49 years. The study also captured all the types of FGM as categorised by WHO. However, there are some potential limitations; the cross-sectional nature of the DHS datasets do not allow for causal relationship in the interpretations. Also, FGM was self reported and some of the questions used to assess the sexual behavioural characteristics maybe under-reported. For instance, there are possibilities of misrepresentation or under-reporting of age at sexual initiation and total number of lifetime sexual partners as well as information on whether those who were married have had a pre-marital sex or the virginity status among young unmarried women due to societal or cultural expectations.

## Conclusions

The study suggests there is no evidence to support the claim that circumcision prevents premarital sex and ensures marital fidelity in Nigeria. While we strongly discourage FGM in all its forms, we assert the need for alternative and acceptable health promoting community measures to address these inherent sexual perceptions toward reducing FGM and improving sexual and reproductive health across population groups.

This may be achieved through community-led and culturally acceptable health education and advocacy campaigns. Further research on this topic and particularly on exploring sexual cultural beliefs and gender dynamics and how these influence FGM across population groups, may be helpful. It is hoped that this study may prompt the needed public health and policy response in Nigeria, and indeed across African countries, towards improved overall sexual and reproductive health.

## Supplementary Information

**Additional file 1: Table S1.** Multivariable log-binomial regression of the association between sexual behaviour and female circumcision among ever married women aged 15–49 years.

**Additional file 2: Table S2.** Multivariable log-binomial regression of the association between sexual behaviour and female circumcision among never married women aged 15–49 years.

## Data Availability

This study used secondary datasets from Measure DHS program, these datasets can be accessed after due permission from the DHS program archive and can be downloaded at https://dhsprogram.com/data/available-datasets.cfm.

## Abbreviations

aRR: Adjusted relative risk
CI: Confidence interval
EAs: Enumeration areas
FCT: Federal Capital Territory
FGM: Female genital mutilation
IQR: Interquartile range
NA: Not applicable
NDHS: Nigerian Demographic Health Survey
SDG: Sustainable development goal
STD: Sexually transmitted disease
WHO: World Health Organization
