# Supplementary material for: Female genital mutilation and sexual behaviour by marital status among a nationally representative sample of Nigerian women
Source: Reprod Health. 2022 Apr 7;19:91. doi: 10.1186/s12978-022-01379-w (PMC8991499; doi:10.1186/s12978-022-01379-w)
Supplement: Supplementary file 1 — Additional file 1: Table S1. Multivariable log-binomial regression of the association between sexual behaviour and female circumcision among ever married women aged 15–49 years. [file 12978_2022_1379_MOESM1_ESM.docx]

**Supplementary Table 1.** Multivariable log-binomial regression of the association between sexual behaviour and female circumcision among ever married women aged 15-49 years.

| **Variable** | **Sexual debut** | **Multiple sexual partner** | **Number of lifetime sexual partner** | **Had STD** | **Pre-marital sex** | **Number of unions** |
| --- | --- | --- | --- | --- | --- | --- |
| Circumcised |  |  |  |  |  |  |
| No | Reference | Reference | Reference | Reference | Reference | Reference |
| Yes | 0.99(0.97-1.00) | **0.80(0.66-0.97)**** | 1.01(0.99-1.02) | **1.18(1.00-1.38)***** | **1.10(1.05-1.15)***** | 1.00(0.99-1.01) |
| **covariates** |  |  |  |  |  |  |
| **Age** |  |  |  |  |  |  |
| 15-19 | Reference | Reference | Reference | Reference | Reference | Reference |
| 20-24 | 1.10(1.04-1.15)*** | 1.33(0.58-3.04) | 1.05(1.03-1.07)*** | 1.66(1.15-2.40)** | 1.45(1.20-1.75)*** | 1.05(1.04-1.07)*** |
| 25-29 | 1.04(0.99-1.09)* | 1.35(0.60-3.05) | 1.10(1.08-1.13)*** | 2.01(1.39-2.90)*** | 1.45(1.21-1.73)*** | 1.09(1.07-1.10)*** |
| 30-34 | 0.98(0.94-1.03) | 2.33(1.02-5.30)** | 1.13(1.11-1.16)*** | 2.28(1.58-3.29)*** | 1.32(1.10-1.58)** | 1.12(1.10-1.14)*** |
| 35-39 | 1.00(0.95-1.03) | 2.50(1.13-5.53)** | 1.13(1.11-1.16)*** | 2.18(1.49-3.19)*** | 1.24(1.04-1.48)** | 1.14(1.12-1.16)*** |
| 40-44 | 0.98(0.93-1.03) | 2.37(1.07-5.27)** | 1.12(1.09-1.14)*** | 2.02(1.38-2.96)*** | 1.16(0.97-1.39)* | 1.16(1.14-1.18)*** |
| 45-49 | 0.96(0.91-1.01)* | 2.40(1.10-5.22)** | 1.10(1.07-1.12)*** | 1.53(1.04-2.24)** | 1.05(0.88-1.26) | 1.17(1.15-1.19)*** |
| **Level of education** |  |  |  |  |  |  |
| No formal education | Reference | Reference | Reference | Reference | Reference | Reference |
| Primary | 1.08(1.05-1.11)*** | 1.96(1.38-2.78)*** | 1.02(1.00-1.04)** | 0.94(0.77-1.15) | 1.13(1.04-1.22)** | 1.02(1.00-1.03)** |
| Secondary | 1.12(1.09-1.15)*** | 1.89(1.32-2.73)*** | 1.04(1.03-1.06)*** | 1.24(0.99-1.54)* | 1.19(1.10-1.29)*** | 0.99(0.98-1.01) |
| Higher | 1.03(1.00-1.06)* | 1.09(0.66-1.80) | 1.01(0.99-1.03) | 1.17(0.89-1.52) | 1.06(0.97-1.16) | 0.97(0.96-0.99)** |
| **Wealth quintiles** |  |  |  |  |  |  |
| Poorest | Reference | Reference | Reference | Reference | Reference | Reference |
| Poorer | 1.04(1.00-1.08)* | 1.14(0.73-1.79) | 1.00(0.98-1.02) | 1.40(1.12-1.74)** | 1.11(0.97-1.27) | 1.00(0.99-1.02) |
| Middle | 1.07(1.03-1.12)*** | 1.38(0.89-2.15) | 1.01(0.98-1.03) | 1.72(1.34-2.20)*** | 1.18(1.02-1.37)** | 1.00(0.99-1.02) |
| Richer | 1.09(1.05-1.13)*** | 0.95(0.60-1.50) | 1.00(0.98-1.03) | 1.50(1.13-2.00)** | 1.17(1.02-1.36)** | 0.99(0.97-1.01) |
| Richest | 1.06(1.02-1.11)** | 0.62(0.38-1.01)* | 0.99(0.97-1.02) | 1.42(1.04-1.93)** | 1.13(0.97-1.31) | 0.96(0.95-0.98)*** |
| **Currently working** |  |  |  |  |  |  |
| No | Reference | Reference | Reference | Reference | Reference | Reference |
| Yes | 0.99(0.96-1.01) | 1.37(1.01-1.82)** | 1.00(0.99-1.02) | 0.90(0.76-1.06) | 1.06(1.00-1.12)** | 1.00(0.99-1.01) |
| **Place of residence** |  |  |  |  |  |  |
| Urban | Reference | Reference | Reference | Reference | Reference | Reference |
| Rural | 0.97(0.95-0.99)*** | 0.80(0.63-1.01)* | 0.99(0.98-1.01) | 0.74(0.61-0.90)** | 0.99(0.94-1.06) | 1.01(1.00-1.02)* |
| **Religion** |  |  |  |  |  |  |
| Christians | Reference | Reference | Reference | Reference | Reference | Reference |
| Muslims | 1.04(101-1.06)** | 0.37(0.24-0.57)*** | 0.92(0.90-0.94)*** | 0.57(0.43-0.75)*** | 0.81(0.75-0.86)*** | 1.01(0.99-1.02)* |
| Others | 1.01(0.93-1.08) | 0.78(0.40-1.51) | 0.90(0.86-0.95)*** | 0.99(0.47-2.10) | 0.62(0.49-0.78)*** | 0.99(0.95-1.03) |
| **Ethnicity** |  |  |  |  |  |  |
| Fulani | Reference | Reference | Reference | Reference | Reference | Reference |
| Hausa | 1.09(1.02-1.15)** | 0.95(0.30-3.03) | 0.97(0.95-0.99)** | 0.85(0.62-1.17) | 0.80(0.61-1.05) | 0.99(0.97-1.02) |
| Igbo | 1.25(1.17-1.33)*** | 1.91(0.62-5.90) | 0.99(0.96-1.03) | 0.96(0.57-1.61) | 1.17(0.91-1.50) | 0.98(0.96-1.01) |
| Yoruba | 1.19(1.11-1.27)*** | 2.29(0.76-6.95) | 0.99(0.95-1.03) | 1.04(0.66-1.62) | 1.16(0.90-1.49) | 0.93(0.90-0.95)*** |
| Others | 1.18(1.11-1.25)*** | 2.33(0.83-6.49) | 1.02(0.99-1.06) | 1.18(0.85-1.64) | 1.34(1.05-1.70)** | 0.96(0.94-0.98)*** |
| **Region** |  |  |  |  |  |  |
| North Central | Reference | Reference | Reference | Reference | Reference | Reference |
| North East | 1.01(0.98-1.05) | 0.54(0.32-0.93)** | 0.95(0.92-0.98)*** | 1.74(1.29-2.36)*** | 0.67(0.57-0.78)*** | 1.01(0.99-1.03) |
| North West | 0.97(0.93-1.01) | 0.30(0.13-0.67)** | 0.92(0.89-0.96)*** | 2.69(1.88-3.83)*** | 0.55(0.42-0.72)*** | 0.98(0.96-0.99)** |
| South East | 0.97(0.94-1.01) | 0.63(0.37-1.05)* | 0.99(0.96-1.03) | 1.72(1.19-2.49)** | 0.92(0.82-1.03) | 0.99(0.97-1.00) |
| South South | 0.96(0.93-0.99)** | 1.11(0.79-1.54) | 1.08(1.05-1.11)*** | 0.43(0.31-0.58)*** | 1.01(0.93-1.09) | 1.01(0.99-1.03) |
| South West | 0.95(0.93-0.98)*** | 1.01(0.72-1.41) | 1.09(1.06-1.12)*** | 0.37(0.26-0.51)*** | 1.09(1.00-1.17)* | 1.02(1.00-1.04)** |
| **Age at marriage** | 1.04(1.03-1.05)*** | 0.99(0.96-1.00)** | 1.01(1.00-1.02) | 0.99(0.98-1.01) | 1.09(1.08-1.10)*** | **0.99(0.99-1.00)***** |

***p<0.001; **p<0.05; *p<0.01
