# Supplementary material for: Female genital mutilation and sexual behaviour by marital status among a nationally representative sample of Nigerian women
Source: Reprod Health. 2022 Apr 7;19:91. doi: 10.1186/s12978-022-01379-w (PMC8991499; doi:10.1186/s12978-022-01379-w)
Supplement: Supplementary file 2 — Additional file 2: Table S2. Multivariable log-binomial regression of the association between sexual behaviour and female circumcision among never married women aged 15–49 years. [file 12978_2022_1379_MOESM2_ESM.docx]

**Supplementary Table 2.** Multivariable log-binomial regression of the association between sexual behaviour and female circumcision among never married women aged 15-49 years.

| **Variable** | **Sexual debut** | **Multiple sexual partner** | **Number of lifetime sexual partner** | **Had STD** | **Pre-marital sex/virginity status** |
| --- | --- | --- | --- | --- | --- |
| Circumcised |  |  |  |  |  |
| No | Reference | Reference | Reference | Reference | Reference |
| Yes | 1.00(0.99-1.01) | 0.99(0.90-1.10) | 1.00(0.98-1.03) | 0.90(0.63-1.29) | 1.02(0.95-1.09) |
| **covariates** |  |  |  |  |  |
| **Age** |  |  |  |  |  |
| 15-19 | Reference | Reference | Reference | Reference | Reference |
| 20-24 | 1.01(0.99-1.02) | 2.54(2.30-2.81)*** | 1.19(1.16-1.22)*** | 4.33(2.63-7.14)*** | 2.54(2.33-2.77)*** |
| 25-29 | 1.02(1.00-1.04)* | 2.83(2.53-3.18)*** | 1.34(1.30-1.38)*** | 5.71(3.41-9.59)*** | 3.02(2.76-3.31)*** |
| 30-34 | 1.01(0.98-1.03) | 2.50(2.08-3.00)*** | 1.48(1.39-1.55)*** | 5.17(2.79-9.60)*** | 3.09(2.77-3.44)*** |
| 35-39 | 0.96(0.91-1.01) | 2.11(1.76-2.53)*** | 1.47(1.45-1.55)*** | 3.98(1.89-8.35)*** | 3.00(2.98-3.68)*** |
| 40-44 | 1.02(0.98-1.06) | 2.14(1.67-2.76) | 1.55(1.45-1.65)*** | 7.70(2.45-24.2)*** | 3.31(2.98-3.68)*** |
| 45-49 | 1.01(0.94-1.08) | 0.89(0.53-1.49) | 1.34(1.22-1.48)*** | 4.76(1.59-14.3)** | 3.29(2.86-3.79)*** |
| **Level of education** |  |  |  |  |  |
| No formal education | Reference | Reference | Reference | Reference | Reference |
| Primary | 0.97(0.94-1.00)* | 1.19(0.87-1.62) | 1.00(0.97-1.04) | 0.88(0.28-2.75) | 1.08(0.87-1.32) |
| Secondary | 1.00(0.97-1.02) | 1.26(0.92-1.73) | 1.01(0.97-1.04) | 1.14(0.39-3.33) | 1.10(0.89-1.36) |
| Higher | 1.02(0.99-1.05) | 1.27(0.93-1.75) | 1.01(0.97-1.05) | 0.90(0.30-2.72) | 1.13(0.91-1.39) |
| **Wealth quintiles** |  |  |  |  |  |
| Poorest | Reference | Reference | Reference | Reference | Reference |
| Poorer | 1.00(0.96-1.03)* | 1.32(1.01-1.72)** | 0.99(0.95-1.03) | 1.55(0.61-3.97) | 1.06(0.89-1.26) |
| Middle | 1.00(0.97-1.03) | 1.29(0.98-1.69)* | 0.97(0.94-1.02) | 1.82(0.72-4.60) | 1.01(0.85-1.20) |
| Richer | 1.01(0.98-1.04) | 1.29(0.98-1.70)* | 1.00(0.96-1.04) | 2.08(0.82-5.26) | 0.99(0.84-1.18) |
| Richest | 1.01(0.98-1.04) | 1.15(0.87-1.52) | 0.99(0.95-1.04) | 2.03(0.79-5.22) | 0.93(0.78-1.10) |
| **Currently working** |  |  |  |  |  |
| No | Reference | Reference | Reference | Reference | Reference |
| Yes | 0.99(0.98-1.00) | 1.24(1.15-1.34)*** | 1.06(1.05-1.08)*** | 1.29(0.91-1.83) | 1.20(1.14-1.27)*** |
| **Place of residence** |  |  |  |  |  |
| Urban | Reference | Reference | Reference | Reference | Reference |
| Rural | 0.98(0.97-0.99)** | 1.08(1.00-1.17)** | 1.01(0.99-1.03) | 1.01(0.73-1.40) | 1.06(1.01-1.12)** |
| **Religion** |  |  |  |  |  |
| Christians | Reference | Reference | Reference | Reference | Reference |
| Muslims | 1.03(1.01-1.05)*** | 0.73(0.62-0.85)*** | 0.95(0.92-0.99)** | 0.95(0.40-2.22) | 0.72(0.64-0.80)*** |
| Others | 0.98(0.88-1.09) | 1.08(1.00-1.17) | 0.98(0.87-1.11) | 3.03(0.35-25.9) | 0.90(0.52-1.56) |
| **Ethnicity** |  |  |  |  |  |
| Fulani | Reference | Reference | Reference | Reference | Reference |
| Hausa | 0.98(0.97-1.00) | 0.76(0.20-2.87) | 0.94(0.89-0.99)** | 0.18(0.03-1.12)* | 0.66(0.24-1.79) |
| Igbo | 0.97(0.94-0.99)** | 3.80(1.42-10.17)** | 1.01(0.95-1.07) | 1.77(0.35-8.89) | 3.11(1.53-6.32)** |
| Yoruba | 1.00(0.98-1.02) | 3.56(1.34-9.45)** | 1.05(0.99-1.12)* | 1.39(0.27-7.22) | 3.01(1.49-6.11)** |
| Others | 0.98(0.96-0.99)** | 3.97(1.51-10.42)** | 1.06(1.01-1.12)** | 2.37(0.47-12.07) | 3.27(1.63-6.57)** |
| **Region** |  |  |  |  |  |
| North Central | Reference | Reference | Reference | Reference | Reference |
| North East | 0.98(0.96-1.02) | 0.86(0.70-1.06) | 0.99(0.95-1.04) | 1.46(0.72-2.99) | 0.70(0.59-0.84)*** |
| North West | 1.01(0.99-1.03) | 0.95(0.58-1.56) | 1.03(0.97-1.10) | 4.38(2.09-9.22)*** | 0.84(0.59-1.18) |
| South East | 0.97(0.95-0.99)** | 1.05(0.87-1.28) | 0.97(0.92-1.02) | 3.29(1.40-7.69)** | 1.04(0.91-1.19) |
| South South | 0.98(0.96-1.00)* | 1.40(1.22-1.61)*** | 1.07(1.04-1.11)*** | 0.92(0.49-1.72) | 1.20(1.09-1.33)*** |
| South West | 0.99(0.98-1.01) | 1.23(1.03-1.47)** | 1.04(0.99-1.08)* | 0.84(0.38-1.89) | 1.11(0.99-1.25)* |

***p<0.001; **p<0.05; *p<0.01
